# Perceptual decision making: drift-diffusion model is equivalent to a Bayesian model

**DOI:** 10.3389/fnhum.2014.00102

**Published:** 2014-02-26

**Authors:** Sebastian Bitzer, Hame Park, Felix Blankenburg, Stefan J. Kiebel

**Affiliations:** ^1^Department of Neurology, Max Planck Institute for Human Cognitive and Brain SciencesLeipzig, Germany; ^2^Bernstein Center for Computational Neuroscience, Charité Universitätsmedizin BerlinBerlin, Germany; ^3^Neurocomputation and Neuroimaging Unit, Department of Education and Psychology, Freie Universität BerlinBerlin, Germany; ^4^Biomagnetic Center, Hans Berger Clinic for Neurology, University Hospital JenaJena, Germany

**Keywords:** perceptual decision making, drift diffusion model, Bayesian models, reaction time, decision variable, parameter fitting, uncertainty

## Abstract

Behavioral data obtained with perceptual decision making experiments are typically analyzed with the drift-diffusion model. This parsimonious model accumulates noisy pieces of evidence toward a decision bound to explain the accuracy and reaction times of subjects. Recently, Bayesian models have been proposed to explain how the brain extracts information from noisy input as typically presented in perceptual decision making tasks. It has long been known that the drift-diffusion model is tightly linked with such functional Bayesian models but the precise relationship of the two mechanisms was never made explicit. Using a Bayesian model, we derived the equations which relate parameter values between these models. In practice we show that this equivalence is useful when fitting multi-subject data. We further show that the Bayesian model suggests different decision variables which all predict equal responses and discuss how these may be discriminated based on neural correlates of accumulated evidence. In addition, we discuss extensions to the Bayesian model which would be difficult to derive for the drift-diffusion model. We suggest that these and other extensions may be highly useful for deriving new experiments which test novel hypotheses.

## Introduction

One of the key questions in neuroscience is how our brain can rapidly categorize its sensory inputs. One approach of addressing this question has been termed perceptual decision making where the typical experiment uses a two-alternative forced choice task to judge differences in perceptual features (e.g., Newsome et al., 1989; Heekeren et al., 2004; Summerfield et al., 2006). The probably best known of these experiments employs the random dot motion task, where subjects decide whether some visually presented dots move either to the left or right (Ball and Sekuler, 1982; Newsome and Paré, 1988). Although this task seems as a severe reduction of the question of how our brain maps its continuous stream of naturalistic, high-dimensional sensory input to one of many different categories, random dot motion experiments have provided a wealth of insightful findings about how perceptual decisions are made (see Gold and Shadlen, 2007, for a recent review).

One key feature of the random dot motion task is that the stimuli are rendered extremely noisy, as compared to input provided under most naturalistic conditions. This high noise level makes the task difficult so that subjects have to sample the sensory input for hundreds of milliseconds to reduce their uncertainty about the stimulus category so that they can commit to a decision. This long observation period is motivated by the experimental aim to delay decisions to differentiate between the mechanisms of evaluating the sensory input and making a decision (Gold and Shadlen, 2007).

The perceptual process has been described by drift-diffusion models where we use the term “drift-diffusion model” to comprise a large variety of similar models (Ratcliff, 1978; Bogacz et al., 2006; Ditterich, 2006; Gold and Shadlen, 2007; Purcell et al., 2010) which all implement the basic mechanism of evidence accumulation with a drift-diffusion process. The underlying assumption commonly is that the brain extracts, per time unit, a constant piece of evidence from the stimulus (drift) which is disturbed by noise (diffusion) and subsequently accumulates these over time. This accumulation stops once enough evidence has been sampled and a decision is made. Drift-diffusion models have been used successfully to quantitatively analyse behavioral data, i.e., reaction times and accuracy (Ratcliff, 1978; Smith and Ratcliff, 2004; Ratcliff and McKoon, 2008). Apart from the typical perceptual decision making paradigms considered here, drift-diffusion models have also been used to explain reaction time distributions in a wide range of other categorization and memory retrieval tasks (see Voss et al., 2013, for review). Furthermore, drift-diffusion models have been used to describe neurophysiological data qualitatively: The mean firing patterns of single neurons, for example, in lateral intraparietal cortex (LIP) of non-human primates exhibit crucial features of the mean trajectories of drift-diffusion models (Ditterich, 2006; Gold and Shadlen, 2007; Purcell et al., 2010).

Another way of modeling the perceptual process is based on Bayesian approaches. There are two different ways of modeling: Firstly, Bayesian schemes have been used for better fitting the parameters of the drift diffusion model to behavioral data, as compared to standard techniques (e.g., Lee et al., 2007; Vandekerckhove et al., 2008; Wiecki et al., 2013). This is not the approach considered here. Rather, we propose a generative model for which we derive the Bayesian inference equations. Similar Bayesian approaches have been used before (Rao, 2004, 2010; Beck et al., 2008; Dayan and Daw, 2008; Brown et al., 2009; Shenoy and Yu, 2011; Denève, 2012; Drugowitsch et al., 2012; Huang et al., 2012). The aim of this paper is to show the exact equivalence between the Bayesian inference equations and the drift diffusion model. The critical difference between the two models is that only the Bayesian model is based on a description of how the sensory input is generated precisely. As we will discuss below, explicitly modeling the sensory input as in the proposed Bayesian model enables a wide range of novel and potentially useful model extensions. More generally, while one appealing feature of drift-diffusion models is their mathematical simplicity, Bayesian models have gained recently a reputation as a potential key to understand brain function in mechanistic-computational terms (Knill and Richards, 1996; Doya et al., 2007; Chater and Oaksford, 2008; Friston, 2010; Pouget et al., 2013). In particular, Bayesian models naturally incorporate the perceptual uncertainty of the observer as a key parameter for explaining behavior.

Specifically, we present a Bayesian model for the two-alternative forced choice task and show that it is equivalent to a pure drift-diffusion model (pDDM) (Bogacz et al., 2006; Wagenmakers et al., 2007), which we call “pDDM” from here on. Conceptually, it has long been known that the pDDM implements the sequential probability ratio test (SPRT) (Bogacz et al., 2006), a procedure which makes statistically optimal decisions. Here, we make the relation to a Bayesian model explicit. The main result is a set of equations, which translate the parameters of the pDDM directly to the parameters of the Bayesian model, and vice versa. This means that one can translate experimentally determined pDDM parameters to the parameterization of a Bayesian model. We demonstrate and cross-validate this translation on a published data set (Philiastides et al., 2011) and show that one can easily cast the drift and diffusion parameters of the pDDM as internal uncertainties of a decision-making observer about the sensory input (Yu and Dayan, 2005; Feldman and Friston, 2010; Dayan, 2012). In addition, using the Bayesian model, we investigate whether there is an experimental way of identifying which computational variable is used by the brain for accumulating evidence. The pDDM postulates that this variable is the so-called log posterior odds while the Bayesian model may also use the posterior or the log posterior. Although all three variables lead to exactly the same decisions, their putative neuronal implementations differ. Identifying which decision variable is used by the brain would cast more light onto the precise neuronal computations the brain performs during the sampling period.

Furthermore, we discuss several potent future developments based on the Bayesian model: For example, it is straightforward to extend the Bayesian model to make specific predictions when using more complex stimuli (as compared to the random dot motion task and similar experimental designs). This may be the basis for future experiments which can test specific and novel hypotheses about the computational mechanisms of perceptual decision making using both behavioral and neuronal data.

## Results

Our main result is the set of equations which relate the parameters of the pDDM and the Bayesian model to each other. To provide the basis for these equations, we describe both models and clarify the conditions under which the equivalence holds. We validate that, in practice, fitting response accuracy and reaction times either using the pDDM, or fitting the Bayesian model directly leads to equivalent results. Finally, we consider three different potential implementations of how the Bayesian perceptual decision making model may be realized in the brain and show that these are unlikely to be differentiated based on current experimental designs.

### Drift-diffusion model

The pDDM (cf. Bogacz et al., 2006; Wagenmakers et al., 2007) is a simple Wiener diffusion process with drift

(1) dy=vdt+sdW

where *y*(*t*) is the diffusion state, *v* is the drift, *s* determines the amount of diffusion and *dW* denotes the standard Wiener process. To show the equivalence we discretize the process to simplify the following analytical treatment:

(2) $$y_{t}-y_{t-\Delta t}=v\Delta t+\sqrt{\Delta t}s\varepsilon_{t}$$

where ε_*t*_ ~ *N*(0,1) is a standard normally distributed noise variable. When used as a model for making perceptual decisions between two alternatives, the diffusion process is bounded above and below by *B* such that decisions are made when |*y*_*t*_| ≥ *B*. If multiple alternatives are considered, a so-called race model may be constructed in which multiple diffusion processes with differing drift race toward a bound in parallel (Bogacz et al., 2006; Gold and Shadlen, 2007).

In summary, the pure drift-diffusion model [pDDM, Equation (2)] accumulates Gaussian-distributed pieces of evidence with mean *v*Δ*t* and variance Δ*ts*^2^ until a bound *B* is crossed. Although these parameters can be fitted to the behavioral responses of a subject, the model does not explain how pieces of evidences are computed from the sensory input, but assumes that this has been done at a lower-level processing stage (Gold and Shadlen, 2007).

### Bayesian model

**Figure 1:**
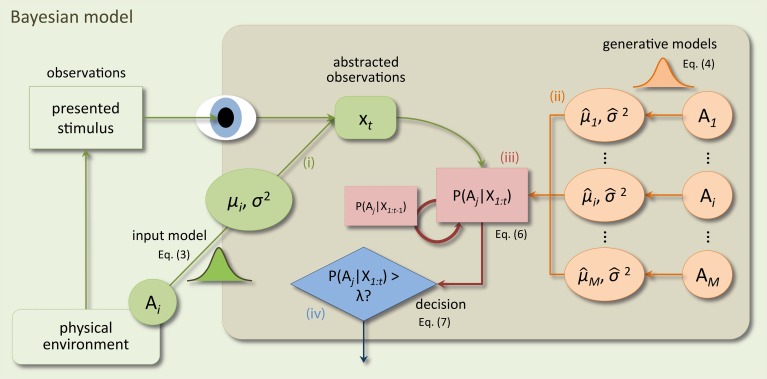
**Schematic of the components of the Bayesian model and how they interact when making decisions at a time point *t* within a trial**. In the physical environment a stimulus is presented by the experimenter and observed by the subject. Components inside the shaded rectangle model internal processes of the subject. Sensory processes in the subject's brain translate the stimulus into an abstract representation *x*_*t*_ (e.g., in a feature space). The input process (i) of the Bayesian model approximates this translation by mapping the stimulus identity (decision alternative *A*_*i*_ out of *M* alternatives) to a value *x*_*t*_ drawn from a Gaussian distribution with mean μ_*i*_ and variance Δ*t*σ^2^. Bayesian inference (iii) recursively computes posterior beliefs over the correctness of a decision alternative given all previous observations *p*(*A*_*i*_|*X*_1:*t*_), *X*_1:*t*_ = {*x*_1_, …, *x*_*t*_} from the previous beliefs *p*(*A*_*i*_|*X*_1:*t*−1_) and the internal generative models (ii). The generative models map decision alternatives to different Gaussian densities which mirror those in the input process (but are allowed to differ) and which are used to compute the likelihood of the current observation *x*_*t*_ under the different alternatives. Decisions are made using a decision policy (iv) based on the computed posterior beliefs and a bound λ.

A critical aspect of a Bayesian model is that it is based on a generative model of concrete sensory observations. To recognize a presented stimulus a Bayesian model compares predictions, based on a generative model, to the observed sensory input. Through Bayesian inference, this comparison leads to belief values indicating how probable it is that the stimulus caused the sensory observations. Note that this is conceptually different from the pDDM where the decision process accumulates random pieces of evidence and there is no explicit representation of raw sensory input. Consequently, a Bayesian model is more complex than the pDDM. As shown in Figure 1 there are four required model components: (i) the generative input process (reflecting the physical environment) which generates noisy observations of stimulus features just as those used in the actual experiment (e.g., dot movements), (ii) internal generative models of the decision maker which mirror the generative input process under the different, hypothesized decision alternatives, (iii) the inference mechanism which translates observations from (i) into posterior beliefs over the correctness of the alternatives using the generative models (ii), and (iv) a decision policy which makes decisions based on the posterior beliefs from (iii). Note that the input process (i) is for neuroscience applications often left unspecified, because experimental data will be used as sensory input. Here, we make the parameterization of the input process explicit, because in typical perceptual decision making experiments, the actual sensory input is not saved or considered relevant, but approximated by its summary measures such as the coherence of random dot motion. For this case, the input process is used as an approximation of the actual sensory input shown to subjects. Figure 1 presents a schematic of the Bayesian model and its four components. For each component, we aimed at choosing the simplest formulation to match the mathematical simplicity of the pDDM. In the following we detail our choices for these four components.

#### Input process

The input process models sensory processes in the brain which translate sensory, e.g., visual, input into simple, noisy feature values that are used for decision making. As we do not know the actual feature values *x*_*t*_ computed by the brain, we presume that they are drawn from a Gaussian distribution whose parameters we will infer from the behavioral data. In particular, we postulate that a feature value (observation) at time *t* is drawn from a Gaussian distribution

(3) $$x_{t}\sim N(\mu_{i},\Delta t\sigma^{2}),$$

if the stimulus that is presented in that trial belongs to decision alternative *i*. The value μ_*i*_ represents the feature value which the brain would extract under perfect noise-free observation conditions. For example, in a task where subjects have to discriminate cars and faces the feature values μ_1_ = −1 and μ_2_ = 1 could represent perfect car-ness and perfect face-ness, respectively. When noise is added to the presented stimuli, feature values can only be extracted imperfectly, which may also be due to physiological noise along the visual pathway in the brain. In this case, the extracted feature values *x*_*t*_ become noisy. We quantify the amount of resulting noise with the variance Δ*t*σ^2^. In the standard random dot motion paradigm (Newsome and Paré, 1988; Gold and Shadlen, 2007) where two opposite directions of motion have to be recognized μ_1_ = −1 would represent one of them (e.g., left) and μ_2_ = 1 the other (e.g., right). Here, the variance Δ*t*σ^2^ would represent the coherence of the dots (greater variance equals smaller coherence) together with physiological noise in the brain.

#### Generative models

For each decision alternative, there is one generative model. We assume that the decision maker aims to adapt its internal generative models to match those of the input process (whose parameters are in principle unknown to the decision maker), but allow for some remaining error between generative models and real input. Consequently, we define the generative model of an abstracted observation *x*_*t*_ for an alternative *A*_*i*_ as Gaussian densities

(4) $$p(x_{t}|A_{i})=N({\hat{\mu }}_{i},\Delta t{\hat{\sigma }}^{2}),$$

where $\hat{\mu }$_*i*_ represents the mean and $\hat{\sigma }$ the internal uncertainty of the decision maker's representation of its observations.

#### Bayesian inference

Bayesian inference prescribes the computations which result in a posterior belief *p*(*A*_*i*_|*x*_*t*_) that alternative *A*_*i*_ is true given observation *x*_*t*_. In perceptual decision making paradigms, where observations *x*_*t*_ arrive sequentially over time, a key quantity is the posterior belief *p*(*A*_*i*_|*X*_1:*t*_) where *X*_1:*t*_ = {*x*_1_, …, *x*_*t*_} collects all observations up to time *t*. This posterior belief can be computed recursively over time using Bayesian inference

(5) $$p(A_{i}|x_{1})=\frac{p(x_{1}|A_{i})p(A_{i})}{\sum_{j=1}^{M}p(x_{1}|A_{j})p(A_{j})}$$

(6) $$p(A_{i}|X_{1:t})=\frac{p(x_{t}|A_{i})p(A_{i}|X_{1:t-1})}{\sum_{j=1}^{M}p(x_{t}|A_{j})p(A_{j}|X_{1:t-1})}$$

where *M* is the number of considered alternatives. This recursive scheme is commonly known as Bayesian updating, but in this particular form it only applies if consecutive observations are independent of each other, as is the standard assumption of drift-diffusion models. Equations (5, 6) state that the posterior belief of alternative *A*_*i*_ is computed by weighting the likelihood of observation *x*_*t*_ under alternative *A*_*i*_ with the previous posterior belief and normalizing the result. This amounts to a comparison of likelihoods across alternatives. At the initial time step the previous belief is the prior belief over alternatives *p*(*A*_*i*_) which can implement biases over alternatives. To implement the absence of a bias, the prior can be chosen to be a uniform distribution.

#### Decision policy

Decisions in the Bayesian model are based on the posterior beliefs *p*(*A*_*i*_|*X*_1:*t*_). Similarly to the threshold mechanism in the pDDM, a decision is made for the alternative with the largest posterior belief when any of the posterior beliefs reaches a predefined bound λ:

(7) $$\underset{i}{\max}p(A_{i}|X_{1:t})\geq \lambda .$$

Alternatively, decisions can be based on transformations of the posterior beliefs. We here consider two other decision variables which are functions of the posterior beliefs (see, e.g., Dayan and Daw, 2008, for a discussion): the log-posteriors

(8) $$\underset{i}{\max}\log p(A_{i}|X_{1:t})\geq \lambda^{\prime }$$

and, in the two-alternative case (*M* = 2), the log posterior odds

(9) $$\left|\log\frac{p(A_{1}|X_{1:t})}{p(A_{2}|X_{1:t})}\right|\geq \lambda^{*}.$$

All three decision policies lead to equivalent decisions (see below). Yet, they prescribe that decisions are based on different quantities (decision variables). Which of them does the brain use? This issue touches on the important question of whether and how the brain represents and computes with probabilities, see e.g., (Denève, 2008; Pouget et al., 2013). One key question is whether the brain represents Bayesian posteriors directly or whether it implements additive accumulation as would be expected for the log posterior odds as used by the pDDM. Principally, their neural correlates should provide an answer, but we show in the results below that, even under ideal experimental conditions, standard perceptual decision making experiments currently cannot discriminate between these decision variables.

### Exact equivalence between bayesian model and the pDDM

Note that the following derivations should not be confused with Bayesian inference methods for parameter fitting of drift-diffusion models (Vandekerckhove et al., 2008; Wiecki et al., 2013). These methods define generative models over the parameters of a drift-diffusion model and infer posterior parameter distributions from decisions which have been observed in an experiment. Here, we do something different: we define a generative model for sensory observations and infer posterior beliefs over the correctness of several decision alternatives. It is, like the pDDM, a model for the internal decision process in the brain where our aim is to show exact equivalence between these two models.

The pDDM has been linked to statistical models in the past. The random walk of the pDDM can be easily related to the SPRT (Bogacz et al., 2006) and the SPRT is known to be Bayes optimal (Wald and Wolfowitz, 1950). Via the SPRT it is then straightforward to see that the Bayesian model and the pDDM must be equivalent: for uniform priors *p*(*A*_*i*_) in the Bayesian model the log posterior odds equal the accumulated log-likelihoods, which can also be used to implement the SPRT:

(10) $$\log\frac{p(A_{1}|X_{1:t})}{p(A_{2}|X_{1:t})}=\sum_{t}\log\frac{p(x_{t}|A_{1})}{p(x_{t}|A_{2})}.$$

We took a direct approach to prove equivalence of the two by showing that sequential updates for the posterior log odds from the Bayesian model implement an update equation of the pDDM form [Equation (2)], see Methods. This resulted into the following relation between parameters of the two models:

(11) $$v=\frac{{\hat{\mu }}_{2}^{2}-{\hat{\mu }}_{1}^{2}}{2{\left(\Delta t\right)}^{2}{\hat{\sigma }}^{2}}+\mu_{i}\frac{{\hat{\mu }}_{1}-{\hat{\mu }}_{2}}{{\left(\Delta t\right)}^{2}{\hat{\sigma }}^{2}}$$

(12) $$s=\sigma \frac{{\hat{\mu }}_{1}-{\hat{\mu }}_{2}}{\Delta t{\hat{\sigma }}^{2}}$$

(13) $$B=\lambda^{*}.$$

Equation (11) states that the drift *v* is a linear function of the feature value of the presented stimulus μ_*i*_ where the slope and offset of the linear function are determined from the parameters of the generative models. Similarly, Equation (12) says that the diffusion *s* is a linear function of the input noise σ with (almost) the same slope as the one for the drift. In other words, drift and diffusion depend on the actual input to the decision maker, but are modulated by its assumptions about the input as expressed in its generative models. Finally, Equation (13) simply states that the pDDM bound equals the bound on the log posterior odds, i.e., that the log posterior odds equal the diffusion state *y*_*t*_.

In addition, for any given Bayesian model in the presented form we can identify a pDDM which makes exactly the same decisions (and at the same time) as the Bayesian model. This is because the decision policy based on the log posterior odds is equivalent to a decision policy operating directly on the posterior under the constraint that

(14) $$\lambda^{*}=\log\left(\frac{\lambda }{1-\lambda }\right)$$

(see Methods for derivation).

### Constraining the bayesian model

While the pDDM has three parameters, the Bayesian model has seven parameters. Therefore, to relate Bayesian model parameters to pDDM parameters, four appropriate constraints must be defined. These constraints can take the simple form of putting absolute values on specific parameters, fixing the ratio of two parameters, or similar. Note that constraints are also required for the pDDM when fitting behavioral data, because the behavioral data only informs about two parameters. Therefore, pDDMs are typically used with the drift fixed to *s* = 0.1 (Bogacz et al., 2006; Vandekerckhove and Tuerlinckx, 2007; Wagenmakers et al., 2007; Ratcliff and McKoon, 2008). The over-parameterization of the Bayesian model does not pose a principled problem, because more parameters may be identified by using either more complex stimulus material or additional data, such as neuronal measurements, see Discussion.

In the next sections, we will motivate and describe two intuitive sets of constraints. By using either of these, one can translate empirically determined parameters of the pDDM to the parameters of the Bayesian model. Although we use below only one of these sets of constraints, we derive both to show that the interpretation of determined parameters given data depends on the chosen constraints.

#### Equal amount of drift for both stimuli

In typical applications of the pDDM it is assumed that the drift for one stimulus is just the negative of the drift for the other stimulus (Wagenmakers et al., 2007; Ratcliff and McKoon, 2008). One reasoning behind this approach is that the log likelihood ratio, on which the pDDM is based, shows this relationship:

(15) $$\log\frac{p(x_{t}|A_{1})}{p(x_{t}|A_{2})}=-\log\frac{p(x_{t}|A_{2})}{p(x_{t}|A_{1})}.$$

However, Equation (11) shows that the drift not only depends on the generative models *p*(*x*_*t*_|*A*_*i*_), but in addition on the distribution of *x*_*t*_ as defined by the input process. Therefore, the absolute amount of drift *v* may, in general, differ for different stimuli.

To map between pDDM and the Bayesian model we also need to ensure that the absolute drift is equal for the two stimuli. There are two different ways to implement this, as shown next.

#### Constraint: symmetric means

The first way to ensure an equal amount of drift for both stimuli is to assume that the means of the input process and the means of the generative models are symmetric around zero, i.e., that their absolute values are equal:

(16) $$\mu_{1}=\mu ,\;\mu_{2}=-\mu ,\;{\hat{\mu }}_{1}=\hat{\mu },\;{\hat{\mu }}_{2}=-\hat{\mu }.$$

Here we denote the common, absolute feature value μ while the actual feature values for the two alternatives remain μ_*i*_ = ± μ where *i* ∈ {1, 2} and indicates a particular alternative. These constraints (constraints 1 and 2) result [by plugging into Equations (11, 12)] in the necessary condition that the signal-to-noise ratios of the pDDM and the input process should be the same (subject to appropriate scaling due to discretization):

(17) $$\frac{v}{s}\Delta t=\frac{\mu_{i}}{\sigma }.$$

Unfortunately, Equations (11, 12) do not allow determining the internal uncertainty $\hat{\sigma }$ from drift and diffusion under the constraints of Equation (16). We, therefore, fix it to an arbitrary value $\hat{\sigma }$ = *a* (constraint 3). Furthermore, either μ (remember that μ_*i*_ = ± μ) or σ needs to be fixed to determine the other variable from Equation (17). We choose to set σ = $\hat{\sigma }$ = *a* (constraint 4) in this example to obtain an explanation based on the means of the input process and generative models only. Using Equations (11, 17), the equations mapping drift *v* and diffusion *s* to the amplitude of the means of the input process μ and the generative models $\hat{\mu }$ are:

(18) $$\mu =\frac{v}{s}\Delta t\sigma$$

(19) $$\hat{\mu }=\frac{1}{2}\frac{s}{\sigma }\Delta t{\hat{\sigma }}^{2}.$$

Thus, with these constraints, the model explains different responses of subjects by different feature values assumed in the input process and the generative models.

#### Constraint: equal means between input process and generative models

The second way to ensure an equal amount of drift for both stimuli is to assume that subjects can determine the means of the two stimuli, as present in the input process, perfectly, i.e.,

(20) $${\hat{\mu }}_{1}=\mu_{1},\;{\hat{\mu }}_{2}=\mu_{2}.$$

(constraints 1 and 2). Under these constraints Equation (11) becomes

(21) $$v=\pm \frac{{\left(\mu_{1}-\mu_{2}\right)}^{2}}{2{\left(\Delta t\right)}^{2}{\hat{\sigma }}^{2}}.$$

where *v* is positive, if stimulus 1 was presented, else negative. Because drift Equation (21) and diffusion Equation (12) then only depend on the difference between means μ_1_ −μ_2_, the means cannot be determined uniquely from drift and diffusion and need to be constrained further. Therefore, we center the means around zero as above, i.e., set μ_1_ = μ and μ_2_ = −μ (constraint 3), without changing the subsequent decisions. We also note that, if the noise standard deviation σ and internal uncertainty $\hat{\sigma }$ are scaled by the same constant, the resulting model is invariant with respect to a scaling of μ [cf. Equations (12, 21) where the common scaling does not change drift and diffusion]. Therefore, without changing the resulting decisions, we can set μ = 1 (constraint 4). The remaining free parameters are the noise variance σ^2^, the internal uncertainty $\hat{\sigma }$ and the bound on the posterior belief λ which can now be determined uniquely from pDDM parameters as

(22) $${\hat{\sigma }}^{2}=\left|\mu_{i(r)}\frac{\mu_{1}-\mu_{2}}{{\left(\Delta t\right)}^{2}v}\right|=\frac{2}{{\left(\Delta t\right)}^{2}v}$$

(23) $$\sigma =\left|\frac{s\Delta t}{\mu_{1}-\mu_{2}}\right|{\hat{\sigma }}^{2}=\frac{1}{\Delta t}\frac{s}{v}$$

(24) $$\lambda =\frac{e^{B}}{1+e^{B}}.$$

These constraints lead to a model which explains response accuracy and reaction time distribution of a subject as the difference between the amount of input noise and the internal uncertainty of the subject while assuming that she has thoroughly internalized the stimulus feature values over a sufficient amount of learning [Equation (20)]. For everyday decisions subjects may have obtained these feature values through general learning about the world, such as when learning about faces and cars. They will then also be able to discriminate, e.g., faces and cars without additional training in a corresponding experiment. Yet, subjects may also refine feature values during an experiment, for example, to better reflect the particular selection of face and car pictures used in the experiment. Then, the constraints in Equation (20) stipulate that the constrained model applies when the feature values have stabilized.

We can use any of the two sets of constraints introduced in the preceding and this section to reparameterize previous results from the pDDM within the present Bayesian model. In the following, we will use the second set of constraints. Our main motivation to choose this specific set of constraints is that it allows us to explain variation in behavior solely with noise and internal uncertainty while the stimulus feature values are kept fixed. As a result, the same uncertainty-mechanism can be applied to dynamic stimuli where the underlying stimulus feature values change with time (see Discussion).

**Figure 2:**
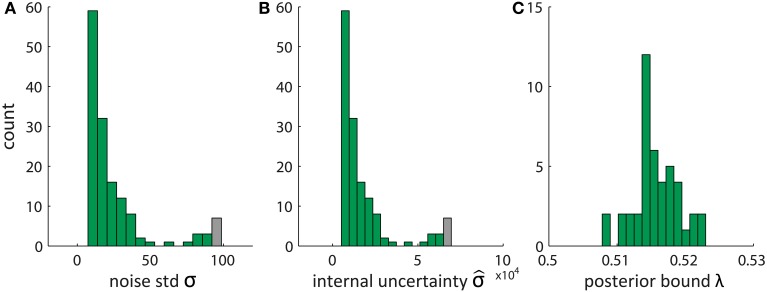
**Estimated parameters of Bayesian model derived from pDDM fits by translating the set of pDDM parameters presented in Wagenmakers et al. (2007)**. **(A)** Noise standard deviation σ, **(B)** internal uncertainty $\hat{\sigma }$, and **(C)** bound on the posterior λ using Equations (22–24). Gray bars summarize the tail of the distribution by also counting data with values larger than the bar edges.

Given the data set of fitted pDDM parameters presented in Wagenmakers et al. (2007) we show in Figure 2 typical values of these parameters identified by applying Equations (22–24). Interestingly, the resulting values of the internal uncertainty $\hat{\sigma }$ and bound λ appear implausible, becauseλ is extremely close to the initial posterior values of 0.5 and $\hat{\sigma }$ is three orders of magnitude larger than the noise standard deviation σ. This is due to an arbitrary common scaling of the parameters *v, s*, and *B* which is resolved by setting the diffusion to *s* = 0.1 in the pDDM (Ratcliff, 1978; Bogacz et al., 2006; Vandekerckhove and Tuerlinckx, 2007; Wagenmakers et al., 2007; Ratcliff and McKoon, 2008). Consequently, we can scale *v, s*, and *B* by a common constant *c* without changing the fit. For *c* > 1 the posterior bound λ increases and the internal uncertainty $\hat{\sigma }$ decreases such that both of them move toward plausible values.

### Inference about parameters using behavioral data

Having established that there is a mapping between the pDDM and the Bayesian model, we tested whether this (theoretical) mapping holds in practice, i.e., when using three different inference methods for a concrete multi-subject data set. Two of these were widely-used inference methods for the pDDM and the third was a method designed for the present Bayesian approach. After fitting with the first two methods we translated the two resulting sets of pDDM parameters to the Bayesian model using Equations (22–24). We expected to find qualitative equivalence and probably minor differences due to the different inference methods.

The data were acquired in a transcranial magnetic stimulation (TMS) experiment which investigated the role of the dorsolateral prefrontal cortex (dlPFC) in perceptual decision making (Philiastides et al., 2011). In the two-alternative forced choice experiment participants had to decide whether a presented noisy stimulus was a face or a car. The (static) pictures of faces or cars were masked with high amounts of noise so that subjects required hundreds of milliseconds to make informed decisions. The authors found that TMS applied over dlPFC made responses slower and less accurate compared to a SHAM stimulation irrespective of stimulus noise level. We use these behavioral data (reaction times and accuracy) to illustrate the analysis and interpretation using the Bayesian model. A more detailed description of the data can be found in Methods.

The three different fitting methods we considered were: (1) EZ: A method proposed by Wagenmakers et al. (2007) which fits the pDDM to error rate, mean and variance of reaction times of correct responses. (2) DMAT: The diffusion model analysis toolbox (DMAT) contains methods presented in Vandekerckhove and Tuerlinckx (2007) which fits various pDDMs to error rates and reaction time distributions of correct and error responses. (3) MCMC: we used a Markov chain Monte Carlo (MCMC) scheme to fit the parameters of the Bayesian model to error rate and reaction time distribution of correct and error responses.

Note that in the pDDM the choice accuracy and reaction times can determine the parameters of the pDDM only up to an arbitrary scaling. The same scaling issue applies to the Bayesian model, but to the internal uncertainty $\hat{\sigma }$ and bound λ because the noise standard deviation σ relates to the fraction of diffusion to drift [Equation (23)] for which a common scaling cancels. This means, on top of using the second set of constraints, for fitting data, there is a fifth constraint necessary. Following the pDDM approach we fix the bound on the posterior λ = 0.7 to prevent misleading variation in parameters due to an arbitrary scaling. We chose this particular value, because it is intermediate between the theoretical extremes of 0.5 and 1 (note that λ is defined on the posterior probability of making a correct decision). In particular, we have observed above (cf. Figure 2) that values of λ close to 0.5 lead to unreasonably large values of the internal uncertainty $\hat{\sigma }$. For the intermediate value λ = 0.7 the internal uncertainty and noise standard deviation σ take comparable magnitudes (see below). Finally, for both pDDM and Bayesian model, we add a non-decision time parameter, *T*_*nd*_, which models delays unrelated to decision making such as due to signal transduction and motor preparation in the brain (Bogacz et al., 2006; Ratcliff and McKoon, 2008). We provide details about the fitting methods in Methods.

**Figure 3:**
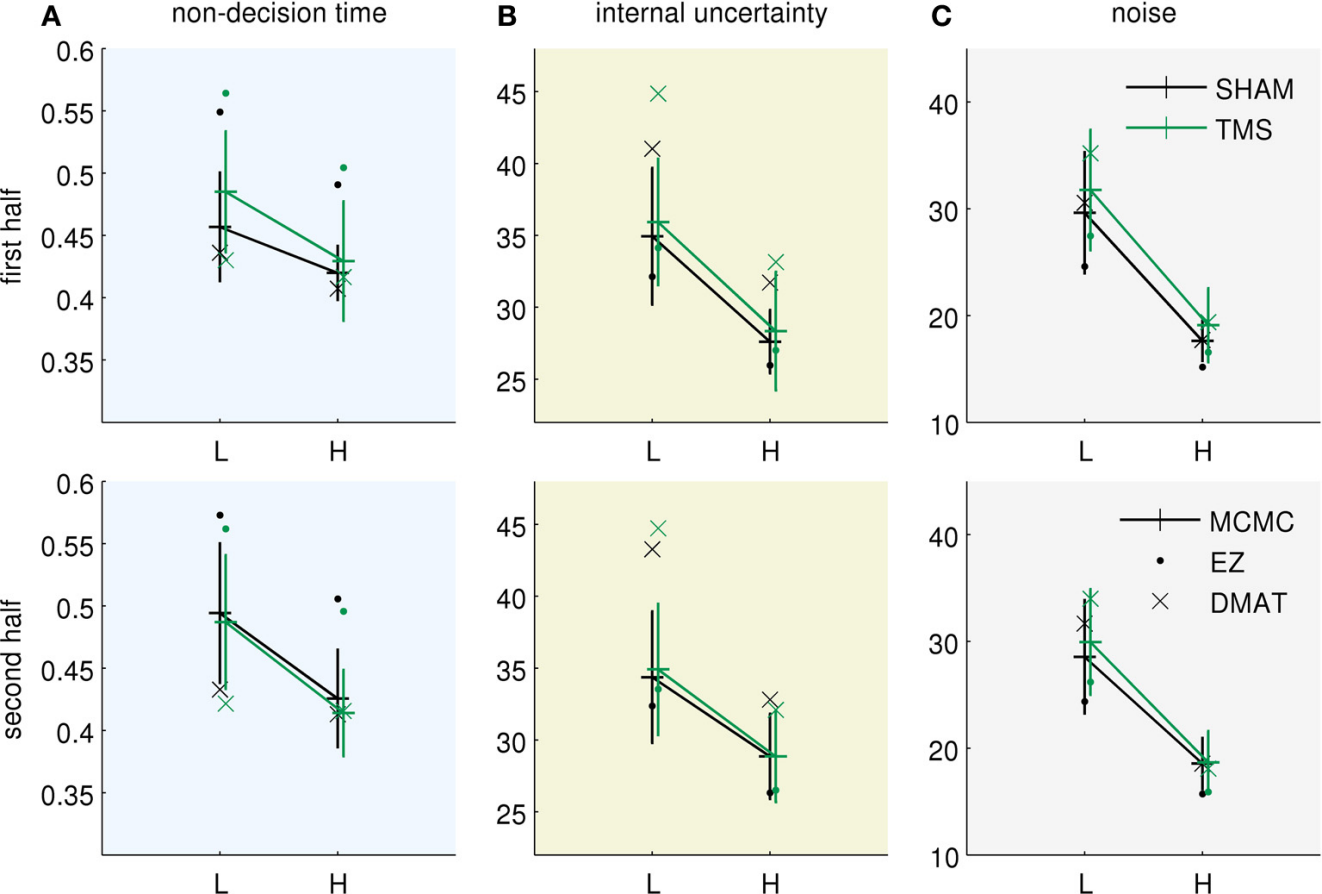
**Bayesian parameters resulting from using three different inference methods (EZ, DMAT, MCMC)**. EZ—EZ method of Wagenmakers et al. (2007), DMAT—fit using diffusion model analysis toolbox of Vandekerckhove and Tuerlinckx (2007) and MCMC—direct Markov chain Monte Carlo fitting of Bayesian model. The parameters of the pDDM fits were translated to the Bayesian model. The data were obtained from Philiastides et al. (2011). L—low evidence (high noise) condition, H—high evidence (low noise) condition, TMS—TMS applied over dlPFC, SHAM—SHAM stimulation instead of TMS. Error bars for MCMC results correspond to double standard deviation of posterior samples. The two rows correspond to the two subsequent halves of the experiment. **(A)** Fits of the non-decision time *T*_*nd*_, **(B)** fits of the internal uncertainty $\hat{\sigma }$, and **(C)** fits of the noise uncertainty σ. All methods allow for qualitatively similar conclusions, see text.

**Figure 4:**
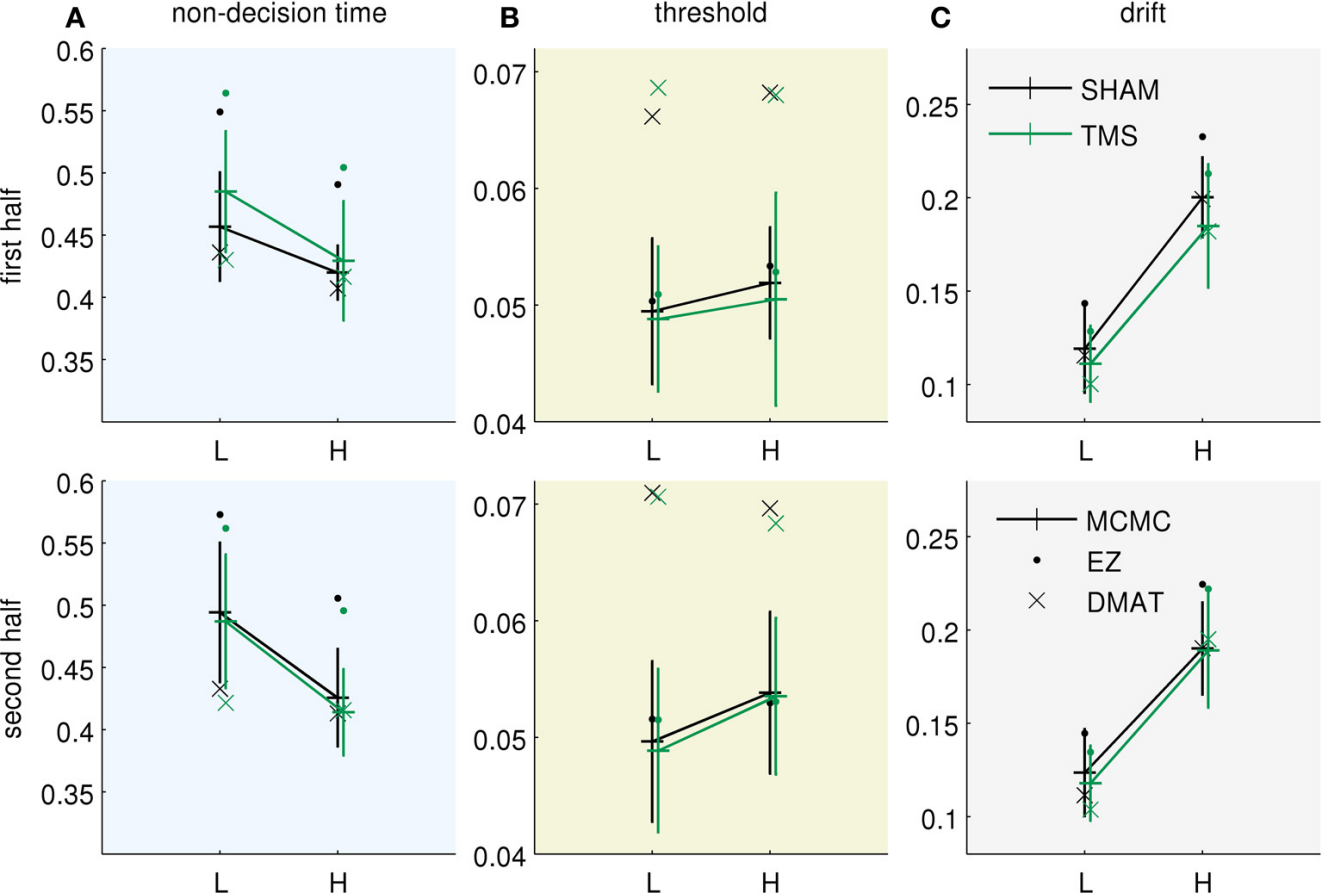
**Equivalent diffusion parameters for the results as presented in Figure 3 (same format)**. The fitted Bayesian model parameters were translated to their equivalent pDDM parameters. **(A)** Non-decision time *T*_*nd*_ (repeated from Figure 3A), **(B)** threshold *B*, and **(C)** drift *v*.

Figure 3 presents the fitted parameter values for all three methods. All of them allow drawing two qualitatively equivalent main conclusions, in terms of the Bayesian model parameterization: (i) The increased uncertainty about the stimuli of the “low-evidence” compared to the “high-evidence” condition is reflected in higher values of both noise standard deviation (Figure 3C) and internal uncertainty (Figure 3B). (ii) The fitted parameters (internal uncertainty $\hat{\sigma }$, and amount of input process noise σ) show an effect of TMS (difference between green vs. black lines in Figures 3B,C), which is reduced from the first half of the experiment (Figure 3, top row) to the second half (Figure 3, bottom row). These findings are equivalent to those presented in the original publication (Philiastides et al., 2011). For completeness we show the equivalent diffusion parameters in Figure 4. These also show the effect reported in the original paper that TMS temporarily reduces drift (Figure 4C). Note, however, that the analysis presented here is based on the pure drift diffusion model applied to data from all subjects. The original analysis in Philiastides et al. (2011) used an extended drift diffusion model to analyse responses of single subjects. Therefore, quantitative differences between the two results should be interpreted with care.

Despite the qualitative consistency of the results across different inference methods there are also minor differences between methods. Compared to MCMC and DMAT, EZ generally inferred a lower amount of noise and internal uncertainty (σ and $\hat{\sigma }$, Figures 3B,C) and compensated for longer reaction times by an increased non-decision time *T*_*nd*_ (Figure 3A). These differences between EZ on one side and DMAT and MCMC on the other may be caused by differences in outlier handling and different representations of the observed reaction time distributions (see Methods).

When comparing MCMC and DMAT, the estimates of internal uncertainty $\hat{\sigma }$ were slightly larger for DMAT (Figure 3B). We found that these differences are due to different choices of the objective function which weigh accuracy and reaction times differently, and to a minor degree, due to differences in the assumed discretization of the two methods (DMAT operates in the continuous limit of the diffusion model where Δ*t* → 0, whereas MCMC works with Δ*t* > 0). DMAT in its standard setting puts a larger weight on reaction times than MCMC, therefore resulting in better fits of accuracy for MCMC. For example, in the “low-evidence” SHAM condition in the first half of the experiment the error rate in the data was 0.19 (plot not shown, see Figure 2A of Philiastides et al., 2011) which was also predicted by the Bayesian model, but the DMAT parameters fit an error rate of 0.15. This underestimation allows DMAT to fit reaction times slightly better than MCMC in some conditions, but at the expense of discarding a larger portion of trials with very long reaction times as outliers.

The MCMC fitting method provides an approximate posterior distribution over the fitted parameters which we used to quantify whether the fitted DMAT or EZ parameter values were closer to those found by MCMC. We implemented this by modeling the posterior MCMC samples with a multivariate Gaussian distribution and evaluating the probability density of the resulting Gaussian at the DMAT and EZ parameter values. In general the estimated density values were very low (<0.0001) for all conditions and halves, indicating that DMAT and EZ parameter sets were rather unlikely under the MCMC posterior (cf. parameter values lying outside of the MCMC error bars in Figure 3). When comparing density values of DMAT and EZ parameters, DMAT parameters were slightly closer to MCMC parameters in the high evidence (low noise) conditions while EZ parameters were closer to MCMC parameters in the low evidence (high noise) conditions.

In summary, these results show that all methods, despite minor differences, provide for a qualitatively equivalent inference.

### Decision variables in the brain

As an example application of the Bayesian model, we now consider the three different decision variables (i) posterior, (ii) log posterior, and (iii) log posterior odds. It is unclear which of the underlying computational mechanisms is used by the brain. This issue touches on the important question of whether and how the brain represents and computes with probabilities (Rao, 2004; Ma et al., 2006; Yang and Shadlen, 2007; Denève, 2008; Fiser et al., 2010; Bastos et al., 2012; Bitzer and Kiebel, 2012; Pouget et al., 2013). One key question is whether the brain represents Bayesian posteriors directly or whether it “only” implements additive accumulation as would be expected for the log posterior odds. The differences are in how exactly the evidence is transformed: The posterior multiplies the evidence for one of the alternatives followed by normalization across alternatives [Equation (6)]. The log posterior corresponds to an inhibited summation of evidence. The simplest form of accumulation is used by the log posterior odds which just sum relative evidences. Note that this formulation is used by the pDDM.

How can one differentiate between these three alternatives experimentally? Behaviorally, all three variables lead to exactly the same decisions and reaction times so that neuronal measurements must be used to address this question. Several neural correlates of decision variables have been identified, e.g., the firing rates of single neurons in monkey parietal cortex (Gold and Shadlen, 2007), or a centro-parietal signal in the human EEG (O'Connell et al., 2012). Here we present simulations using the Bayesian model which suggest that a naive approach of using typical perceptual decision making experiments would most likely not allow discriminating between the three different decision variables.

**Figure 5:**
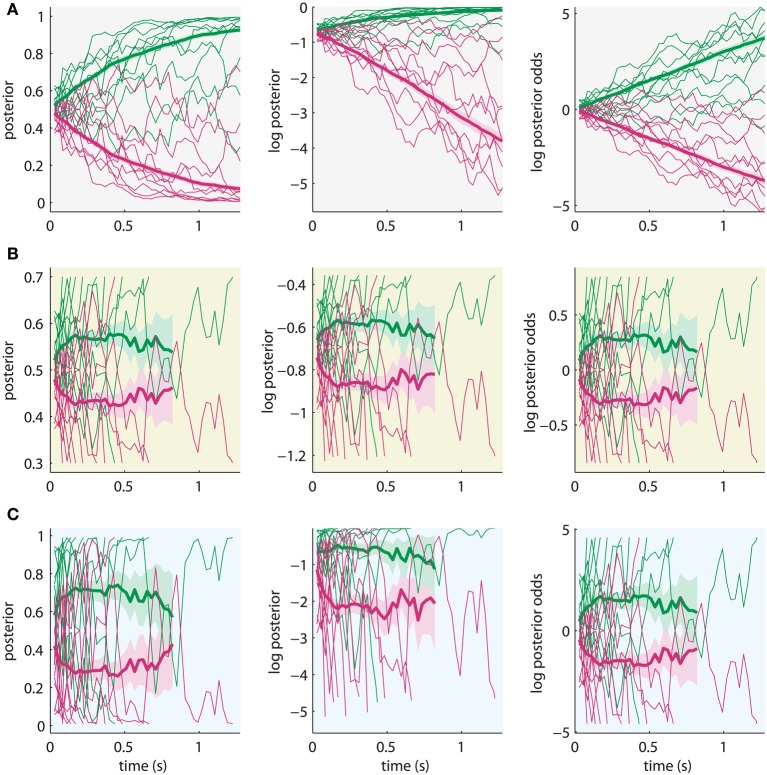
**Sample trajectories from 10 trials (thin lines) and average trajectory over 300 trials (thick lines) of the different decision variables (left: posterior, middle: log posterior, right: log posterior odds) for the same noisy input**. Shading around average trajectories indicates their standard error. Green lines: correct alternative, red lines: incorrect alternative. The sample trajectories were simulated using the Bayesian model with parameters which fit behavioral data of the experiment in Philiastides et al. (2011). **(A)** All trajectories are shown for the maximum length of a trial, ignoring the bounds (λ for posteriors, log λ for log posteriors and λ^*^ for log posterior odds). Decision variables can be differentiated based on asymptotic behavior. **(B)** Sample trajectories (thin lines) are only plotted until crossing a bound and averages (thick lines) are based only on data points before crossing the bound. Rescaling is applied to account for unknown scaling of neural correlates of the decision variables. Note that standard errors of averages increase with time because fewer trials contribute data at later time points. Average trajectories stop when less than 12 trials contribute data. Decision variables cannot be distinguished. **(C)** Simulation with parameters which still produce the same decisions, but with very high bound (λ = 0.99). Decision variables may be distinguished, but effects are minor.

The potential difference, which may be accessible with neuronal measurements, is the asymptotic behavior of the decision variables when reaching high certainty for the decision. Figure 5 shows example trajectories of the three decision variables for exactly the same noisy input. We simulated the trajectories using the Bayesian model with the posterior means inferred from the “high-evidence” SHAM condition in the first half of the experiment (σ = 18.1, $\hat{\sigma }$ = 28.6, see Figure 3).

Figure 5 shows that the posterior converges toward 1 and 0 (Figure 5A, left), the log posterior converges to 0 at the top and is unbounded at the bottom (Figure 5A, middle), and the log posterior odds are unbounded both at the top and bottom (Figure 5A, right). These differences could be used to discriminate decision variables based on measurements of their neural correlates. However, these plots ignore the bounds on the decision variables, which model that the subject's brain stops accumulating when committing to a decision. Thus, these trajectories cannot be observed experimentally. When we apply the bounds from the quantitative analysis of the (Philiastides et al., 2011) data (λ = 0.7), the trajectories become indistinguishable after appropriate scaling (Figure 5B). The difference in absolute amplitude across decision variables cannot be used for differentiation, because there may be an unknown scaling in the mapping of these variables to their neuronal expression. Therefore, it is unlikely that, under the conditions of the typical experiment, decision variables can be discriminated based on neural correlates, because the accumulation of evidence stops considerably before the decision variables converge toward their asymptotes.

This result depends on the specific choice for the bound which we arbitrarily fixed to λ = 0.7 for fitting the data of Philiastides et al. (2011). The inability to differentiate decision variables may, thus, be due to our modeling choices and not an intrinsic property of the decision mechanisms. If we had chosen the much higher bound λ = 0.99 during fitting, which is already close to the asymptote of the posterior at 1, the internal uncertainty would have been $\hat{\sigma }$ = 12.3 instead of $\hat{\sigma }$ = 28.6 (note the arbitrary scaling between $\hat{\sigma }$ and λ). The responses predicted by the model with these parameters would be the same, but individual steps in the decision variables would be larger and would partially cancel the effect of the increased bound. Consequently, even with such a high bound, the asymptotic behavior of the decision variables is not apparent (Figure 5C) such that it appears that they cannot be discriminated based on neural correlates of accumulated evidence.

More light may be shed on this issue by setting up experiments where subjects are made to use larger bounds at the same noise level of the stimuli and same internal uncertainty. For example, for λ = 0.99 without changing any of the other parameters the trajectories of the decision variables would closely resemble those of Figure 5A, because decisions become very slow (plots not shown).

## Discussion

We have presented a Bayesian model with which behavioral data from perceptual decision making experiments can be analyzed. The main result of this paper is that the Bayesian model is equivalent to the pDDM which underlies many widely used models of perceptual decisions (Bogacz et al., 2006; Gold and Shadlen, 2007; Wagenmakers et al., 2007; Ratcliff and McKoon, 2008; Purcell et al., 2010). Conceptually, the Bayesian model allows interpreting the perceptual decision process in terms of predictive coding which postulates that decisions are based on a comparison of predicted and observed sensory input. We have shown that perceptual decision making behavior can be explained by varying amounts of sensory noise and the decision maker's uncertainty. A major advantage of the Bayesian model is that it can easily be extended to incorporate prior knowledge about the decision process, see below.

We derived equations which translate parameters obtained by fitting the pDDM to parameters of the Bayesian model, and vice versa. In this way, already published pDDM results can be interpreted in terms of the Bayesian model, for example, in terms of uncertainties perceived by the decision maker. In addition, we showed that this equivalence also holds in practice, for a previously published behavioral study (Philiastides et al., 2011), by comparing the inferred parameters of the Bayesian model with the translated versions of the fitted parameters of two different, well-established inference schemes for the pDDM (Vandekerckhove and Tuerlinckx, 2007; Wagenmakers et al., 2007). As a further example application of the Bayesian model we addressed the question of which decision variable (posterior, log-posterior, or log posterior odds) the brain may use and pointed to experimental conditions which may allow investigating this question based on neural measurements.

The particular Bayesian model we chose is based on one of the simplest models that can be used to describe perceptual decisions. In particular, we assumed that the stimuli are represented by a single feature value with additive Gaussian noise. This parsimonious choice is owed to the lack of better knowledge about the stimulus representations which are used by the brain to make perceptual decisions. This specific representation as a single feature value applies to a wide range of stimuli (e.g., left vs. right motion in random dot stimuli, or faces vs. cars in Philiastides et al., 2011).

The present Bayesian model is related to previous Bayesian models of decision making. For example, Dayan and Daw (2008) described Bayesian decision models in the context of partially observable reinforcement learning problems and suggested Gaussian models for sequential inference in perceptual decision making but without establishing the details of the equivalence to the pDDM. Daunizeau et al. (2010) inferred parameters of a Bayesian model very similar to the one presented here from reaction times in a learning task, but the model was not aimed at perceptual decision making since the process of how noisy evidence is accumulated within a single trial was not modeled. A Bayesian model closely related to the present model was presented in Drugowitsch et al. (2012). This model consists of a Gaussian input process, Gaussian generative models, computes the posterior beliefs and has a decision policy based on them. Drugowitsch et al. (2012) further showed that the posterior beliefs can be expressed as a function of the accumulated samples from the input process and elapsed time. Our approach has the advantage that the equations which relate the parameters of the two models [Equations (11–14)] can be derived in a straightforward fashion.

To establish the translation of pDDM parameters to those of the Bayesian model, we had to impose additional constraints on the Bayesian model parameterization, because the Bayesian model, in its full version, has seven parameters, but the pDDM has only three. We considered two different sets of constraints, which made different assumptions about parameters or parameter ratios. For fitting a multi-subject data set (Philiastides et al., 2011), we used one of these constraint sets where the decision maker optimally represents the means of the stimulus features. This set of constraints leaves the uncertainty of the stimulus (amount of noise) and the internal uncertainty of the generative stimulus models of the decision maker as free parameters. Although it may be tempting to interpret these variances as reflecting two different sources of noise in the brain, one external, sensory noise and one internal, decision noise, this interpretation would be misleading: the internal uncertainty rather quantifies the decision maker's expectation about the variance of the only noise source in the model, the input process. This expectation modulates (by a gain factor) how the decision maker processes the input [see, e.g., Equations (11, 12)], but the pieces of evidence which result from this computation are accumulated perfectly. Incidentally, experimental evidence for almost perfect accumulation in humans and rats has been reported recently (Brunton et al., 2013). Using the model, it would be interesting to investigate discrepancies between the actual noise variance and the decision maker's expectation about this variance as resulting from fits of these parameters to responses. However, the use of constraints on other key parameters (see section Constraint: Equal Means Between Input Process and Generative Models) currently does not allow this interpretation. The behavioral data (choices and reaction times) enable the identification of two parameters only; if the model can be extended to include other data sources, e.g., neuronal data, more parameters may be resolved and one may investigate how well individual decision makers estimate their input distributions for a given task.

The equivalence of pDDM and Bayesian model means that their results can be easily translated between parameterizations. We presented an example where we fitted the pDDM to data and translated the determined parameters to the Bayesian model. This approach has the advantage that the previously suggested and highly efficient methods for fitting the diffusion model (Vandekerckhove and Tuerlinckx, 2007; Wagenmakers et al., 2007; Ratcliff and McKoon, 2008) can be used for the Bayesian model as well. In addition, we also demonstrated a fitting technique for the Bayesian model based on a MCMC approach. The MCMC method is computationally intensive, as individual experiments are repeatedly simulated to estimate corresponding summary statistics of the data. However, the advantage of the MCMC method is its flexibility. For example, it can be applied with any set of constraints and can be used for fitting extended Bayesian models (see below) for which there is no correspondence to a pDDM.

The Bayesian model can be used to investigate novel experimental questions. For example, we have shown that three different decision variables can be derived from the posterior probabilities of the decision alternatives. All three decision variables can lead to the same decisions and reaction times and we have shown that under typical experimental conditions it is hard to discriminate them even based on neural correlates of accumulated evidence. Consequently, to investigate which of the decision variables may be implemented in the brain we proposed to manipulate the bound subjects use to make decisions. It has been suggested that the bound depends on the reward of making a correct, or the cost of making a wrong choice (Wald, 1947; Gold and Shadlen, 2002; Dayan and Daw, 2008; Rao, 2010; Drugowitsch et al., 2012; Huang et al., 2012). Manipulating the rewards associated with choices may, therefore, lead to the desired increases of the bound in different experimental conditions and a discrimination of the three decision variables may be possible. However, note that the perceptual processes considered here may not be easily modifiable by task conditions, but may be rather set in their ways. A recent study showed that rats, even when given reward, cannot deliberately lengthen their accumulation period for specific tasks (Zariwala et al., 2013).

### Extensions

One key advantage of Bayesian models is the ease of including prior information in an analysis. This enables the incorporation of additional, even uncertain information about the involved processes and model components. We here discuss such possible extensions of the present Bayesian model.

In the description above [Equations (3–9)] we did not specify the number of decision alternatives to highlight that the Bayesian model applies equally to decisions involving an arbitrary number of alternatives. For two alternatives the Bayesian model implements the SPRT, just as the diffusion model (Bogacz et al., 2006). For multiple alternatives the Bayesian model implements a “multihypothesis sequential probability ratio test” (MSPRT) (Dragalin et al., 1999), but there is no longer a direct correspondence to a simple diffusion model in the form of Equation (2). Instead, similar models based on inhibition between diffusive integrators have been proposed to implement the MSPRT (Bogacz and Gurney, 2007; Ditterich, 2010; Zhang and Bogacz, 2010). These models, therefore, should be (approximately) equivalent to the Bayesian model, but the exact relations between parameters of the models are unknown.

Ratcliff's diffusion model (Ratcliff, 1978) includes several extensions of the pDDM in Equation (1) which allow for better fits of reaction time distributions (see Ratcliff and McKoon, 2008 for a review). These extensions can be easily incorporated into the Bayesian model. The first extension is adding a bias which leads to preference of one alternative over the other. Subjects have been shown to use such a bias when making perceptual decisions (e.g., Mulder et al., 2012, and references therein). In the Bayesian model a bias can be realized by using non-uniform priors over alternatives [*p*(*A*_*i*_) in Equation (5), see Methods section Equivalence of Bayesian Prior and pDDM Bias]. The remaining extensions implement across-trial variability in bias, drift and non-decision time which can equally be implemented in the Bayesian model by considering the relations between parameters. For example, in Ratcliff's diffusion model drift *v* is assumed to be drawn randomly from a Gaussian distribution at the start of a trial. Using, e.g., Equations (22, 23) we can determine the particular variability in σ and $\hat{\sigma }$ which would replicate the distribution of *v* in the Bayesian model.

A powerful way to extend the Bayesian model is to consider decision theory. As mentioned above, the bound can implement optimal decisions reflecting the reward structure of the task (Wald, 1947; Gold and Shadlen, 2002; Dayan and Daw, 2008; Solway and Botvinick, 2012; Summerfield and Tsetsos, 2012). For example, Drugowitsch et al. (2012), have investigated how experimentally observed decisions can be explained by time-varying, internal costs of the subjects. By allowing for time-varying, reward- and cost-dependent bounds they fitted reaction time distributions better than with the pDDM. An equivalent analysis would apply to the Bayesian model presented here: By assuming rewards for correct responses, costs for error responses and time-varying costs for sampling new observations time-varying bounds can be derived which lead to new predictions for the quality and timing of decisions.

If more information about the stimulus features and their encoding in the brain is available, this information can in principle be incorporated into the Bayesian model by appropriately adapting the definitions of input process [Equation (3)] and generative models [Equation (4)]. For example, if stimulus features are believed to be better described by a Poisson distribution (cf. Gold and Shadlen, 2001), the input process can be adapted correspondingly. The resulting model would clearly not map to a Gaussian diffusion model as defined by Equation (1). It is further interesting to note that the equivalence between Bayesian model and pDDM only holds as long as the generative models use equal variances for the two alternatives ($\hat{\sigma }$_1_ = $\hat{\sigma }$_2_). For unequal variances the diffusion becomes non-Gaussian and the pDDM does not apply anymore.

Finally, the Bayesian model allows for the important extension that the average amount of evidence can change within a trial. In particular, Equation (6) only applies under the assumption that individual observations *x*_*t*_ are independent from each other, but a similar recursive formula can be derived for stimuli whose mean feature values μ vary predictably within a trial, i.e., for general dynamic stimuli such as biological motion (Bitzer and Kiebel, 2012). In the pDDM time-varying average evidence translates into a time-dependent drift. Similar models have been used to discriminate between decision mechanisms (Cisek et al., 2009; Tsetsos et al., 2012; Brunton et al., 2013) based on simple, pulsed stimuli. In the Bayesian model, one could use dynamic generative models which represent biological motion stimuli so that the evolution of the decision variable in response to a specific, continuous stimulus can be predicted. We will consider this extension in a future publication.

In summary, we have presented a Bayesian model and its equivalence with the pDDM. As we have shown, this provides for deeper insight into the underlying assumptions made by models of perceptual decision making. In practice, as we illustrated on a multi-subject data set, parameters can be translated between the pDDM and the Bayesian model in a straightforward fashion. The key advantage of the Bayesian model is that it can be extended easily to model experimental data different from the prototypical random dot motion experiment. In particular, these extensions may allow for new insights into the mechanisms underlying perceptual decision making in the future.

## Methods

### Equivalence proof

Here we derive the equivalence of the diffusion model defined by Equation (2) and the Bayesian model defined by Equations (3–9).

#### Equivalence of bayesian decision policies

First, we show that the three decision policies [Equations (7–9)] make the same decisions when the bound is appropriately chosen. It becomes clear immediately that the policy on the posterior [Equation (7)] and the log posterior [Equation (8)] make the same decisions, if the bound on the log posterior equals the logarithm of the bound on the posterior, i.e., λ′ = log λ (the log is applied to both sides). To establish equivalence between the posterior and the log posterior odds [Equation (9)] we first assume without loss of generality that stimulus 1 was presented such that the log posterior odds tend to be positive and the absolute value bars can be dropped in Equation (9). It follows that the posterior for decision alternative 1 is the largest such that we can take *i* = 1 in Equation (7). We further define *z*_*i,t*_ = *p*(*A*_*i*_|*X*_1:*t*_) and the unnormalized posterior values as $\tilde{z}$_*i,t*_ = *p*(*x*_*t*_|*A*_*i*_)*p*(*A*_*i*_|*X*_1:*t*−1_). Then the decision policy on the posterior is

(25) $$\frac{{\tilde{z}}_{1,t}}{{\tilde{z}}_{1,t}+{\tilde{z}}_{2,t}}\geq \lambda$$

(26) $$\Leftrightarrow \log{\tilde{z}}_{1,t}-\log\left({\tilde{z}}_{1,t}+{\tilde{z}}_{2,t}\right)\geq \log\lambda .$$

By using the log-sum-exp trick (Murphy, 2012, p. 86) and using the assumption that $\tilde{z}$_1,*t*_ > $\tilde{z}$_2,*t*_, because stimulus 1 was presented, it follows that

(27) $$\log{\tilde{z}}_{1,t}-\log\left({\tilde{z}}_{1,t}+{\tilde{z}}_{2,t}\right)\geq \log\lambda$$

(28) $$\begin{array}{l} \Leftrightarrow \log{\tilde{z}}_{1,t}-\log{\tilde{z}}_{1,t}-\log(\exp(0)\\ \;+\exp\left(\log{\tilde{z}}_{2,t}-\log{\tilde{z}}_{1,t}\right))\geq \log\lambda \end{array}$$

(29) $$\Leftrightarrow {\left(1+\exp\left(\log{\tilde{z}}_{2,t}-\log{\tilde{z}}_{1,t}\right)\right)}^{-1}\geq \lambda$$

(30) $$\Leftrightarrow \log\left(\frac{1}{\lambda }-1\right)\geq \log{\tilde{z}}_{2,t}-\log{\tilde{z}}_{1,t}$$

(31) $$\Leftrightarrow \log{\tilde{z}}_{1,t}-\log{\tilde{z}}_{2,t}\geq \log\left(\frac{\lambda }{1-\lambda }\right)=\lambda^{*}$$

where we have used

(32) $$\log\frac{p(A_{1}|X_{1:t})}{p(A_{2}|X_{1:t})}=\log{\tilde{z}}_{1,t}-\log{\tilde{z}}_{2,t}$$

in the last step. This shows that the decision policy defined on the posterior with bound λ equals the decision policy defined on the log posterior odds, if the bound λ^*^ relates to λ via Equation (14).

#### Equivalence of accumulation mechanisms

We now show that there is a simple recursive formula for the log posterior odds which provides the equivalence relations presented in Equations (11–13).

Let us define

(33) $$q_{t}=\log\frac{p(A_{1}|X_{1:t})}{p(A_{2}|X_{1:t})}=\log z_{1,t}-\log z_{2,t}$$

A recursive formula for *q*_*t*_ results from using the recursive Bayes update equation [Equation (6)]:

(34) $$\begin{array}{l} q_{t}=\log z_{1,t}-\log z_{2,t}\\ \;=\log p(x_{t}|A_{1})+\log z_{1,t-\Delta t}-\log Z_{t}\\ \;-\log p(x_{t}|A_{2})-\log z_{2,t-\Delta t}+\log Z_{t}\\ \;=\log p(x_{t}|A_{1})-\log p(x_{t}|A_{2})+q_{t-\Delta t} \end{array}$$

where *Z*_*t*_ = *p*(*x*_*t*_|*A*_1_)*z*_1,*t*−Δ*t*_ + *p*(*x*_*t*_|*A*_2_)*z*_2,*t*−Δ*t*_ is the normalization constant of the posterior.

By plugging the definition of the generative models [Equation (4)] into Equation (34) one obtains

(35) $$\begin{array}{l} q_{t}=-\log Z-\frac{1}{2\Delta t{\hat{\sigma }}^{2}}{\left(x_{t}-{\hat{\mu }}_{1}\right)}^{2}+\log Z\\ \;+\frac{1}{2\Delta t{\hat{\sigma }}^{2}}{\left(x_{t}-{\hat{\mu }}_{2}\right)}^{2}+q_{t-\Delta t}\\ \;=\frac{1}{2\Delta t{\hat{\sigma }}^{2}}\left(2\left({\hat{\mu }}_{1}-{\hat{\mu }}_{2}\right)x_{t}+{\hat{\mu }}_{2}^{2}-{\hat{\mu }}_{1}^{2}\right)+q_{t-\Delta t}\\ \;=\hat{v}+\hat{s}x_{t}+q_{t-\Delta t} \end{array}$$

with

(36) $$\hat{v}=\frac{{\hat{\mu }}_{2}^{2}-{\hat{\mu }}_{1}^{2}}{2\Delta t{\hat{\sigma }}^{2}}\text{ and }\hat{s}=\frac{{\hat{\mu }}_{1}-{\hat{\mu }}_{2}}{\Delta t{\hat{\sigma }}^{2}}.$$

Using the definition of *x*_*t*_ from the input process [Equation (3)] one can write

(37) $$\begin{array}{l} q_{t}-q_{t-\Delta t}=\hat{v}+\hat{s}\left(\mu_{i(r)}+\sqrt{\Delta t}\sigma \varepsilon_{t}\right)\\ \;=v\Delta t+\sqrt{\Delta t}s\varepsilon_{t} \end{array}$$

where now *v* and *s* are defined by Equations (11) and (12), respectively, and it can be seen that the resulting update equation equals that of the pDDM [Equation (2)].

#### Equivalence of bayesian prior and pDDM bias

The Bayesian model defines a prior probability over alternatives: *p*(*A*_*i*_). In the two-alternative case of the pDDM this prior directly translates to an offset *y*_0_ of the initial diffusion state. To show this we consider the log posterior odds at the first time point:

(38) $$q_{1}=\log\frac{p(A_{1}|x_{1})}{p(A_{2}|x_{1})}=\log p(x_{1}|A_{1})-\log p(x_{1}|A_{2})+y_{0}$$

where we have defined

(39) $$y_{0}=\log p(A_{1})-\log p(A_{2})=\log\frac{p(A_{1})}{p(A_{2})}.$$

Because the prior over all alternatives needs to sum to 1, we can replace *p*(*A*_1_) = *p*_0_ and *p*(*A*_2_) = 1−*p*_0_ such that we arrive at the following relationship between Bayesian prior and diffusion bias:

(40) $$y_{0}=\log\frac{p_{0}}{1-p_{0}}$$

(41) $$p_{0}=\frac{e^{y_{0}}}{1+e^{y_{0}}}$$

which simply replicates the relation between the bounds of the Bayesian model (λ) and the pDDM (*B*).

### Data of (Philiastides et al., 2011)

The experiment used a two-factorial design consisting of factors “evidence level” (high vs. low) and “stimulation” (TMS vs. SHAM), respectively (Philiastides et al., 2011). Each subject performed two blocks of 400 two-alternative forced choice trials; one with TMS and the other with SHAM stimulation applied before the beginning of a block. The order of TMS and SHAM was counterbalanced among subjects. In each block, trials with different evidence levels (high/low) were interleaved. In the analysis the 400 trials in each block were divided into the first and second half (200 trials each). For each of the halves, trials were further divided into those with high and low evidence. In sum, each of the four experimental conditions (a combination of a particular level from both the “evidence level” and “stimulation” factors) had 100 trials per half and there were 800 trials per subject in total. Philiastides et al. (2011) tested 11 subjects whose data (response and reaction time) we pooled into one big data set with 1100 trials per condition per half of a block. We fitted the Bayesian model (see below) independently to each of these eight data sets (2 × 2 × 2: TMS/SHAM stimulation × high/low evidence × first/second half). As in Philiastides et al. (2011) trials in which no decision had been reported within 1.25 s were declared as outliers and removed from further analysis. For further details about the subjects, design, and acquisition see Philiastides et al. (2011).

### Fitting methods

We will first sketch the methods based on fitting the pDDM and then describe the method used to fit the Bayesian model.

#### EZ

Wagenmakers et al. (2007) presented the EZ-diffusion model to provide a relatively simple way of fitting a drift-diffusion model to accuracy and reaction times. They showed that the free parameters of the EZ-diffusion model (drift *v*, bound *B*, and non-decision time *T*_*nd*_) can directly be determined from the proportion of correct decisions, the mean reaction time for correct decisions (MRT) and the variance of reaction times for correct decisions (VRT). This method, therefore, assumes that the summary statistics MRT and VRT are sufficient to describe the observed reaction time distribution. Furthermore, the method assumes that MRT and VRT are noiseless statistics describing the reaction time distribution generated by the diffusion model in continuous time [Equation (1), or Δ*t* → 0 in Equation (2)]. Although outlier trials, for example, defined by too large reaction time, may be removed before estimating MRT and VRT from experimental data, the reaction time distribution predicted by the diffusion model is implicitly allowed to exhibit large reaction times which exceed the outlier definition. This discrepancy between data preprocessing and modeling may lead to minor distortions in the fitted parameter values when compared to a method that considers outliers during RT distribution modeling (see next section).

We use the implementation of the fitting equations in ezdiff.m of DMAT (see below) to obtain values for *v, B*, and *T*_*nd*_. Subsequently, we find a common scaling of drift *v*, diffusion *s*, and bound *B* which implements the constraint in the Bayesian model that the bound on the posterior is λ = 0.7. The corresponding scaling constant *c* is

(42) $$c=\log\left(\frac{\lambda }{1-\lambda }\right)/B.$$

Note that the EZ-diffusion model is not parameterized directly with the bound *B*, but instead determines a boundary separation *a* with *B* = *a*/2, because the initial state of the drift-diffusion process is set to *a*/2. We determine the translated parameters of the Bayesian model from the scaled diffusion parameters using Equations (22–24).

#### DMAT

The publicly available diffusion model analysis toolbox (DMAT, http://ppw.kuleuven.be/okp/software/dmat/) provides Matlab routines for fitting extended pDDMs to accuracy and reaction time data (Vandekerckhove and Tuerlinckx, 2007). In contrast to EZ this method uses quantiles or histograms to represent reaction time distributions for both correct and error responses. It therefore allows for more flexible representations of the reaction time distributions which can capture more variation in reaction times than EZ. Also, other than for the EZ method, there is no closed-form solution which provides best fitting diffusion parameters in the DMAT formulation. Instead, in DMAT parameters are fitted using non-linear optimization which maximizes a multinomial likelihood function based on numerical estimates of the cumulative reaction time distribution predicted by the extended pDDM (Vandekerckhove and Tuerlinckx, 2007). Similar to EZ, these predictions are determined for the continuous limit of the pDDM (Δ*t* → 0) via the first passage time distributions for a bounded drift-diffusion process (Ratcliff, 1978; Vandekerckhove and Tuerlinckx, 2007).

DMAT provides highly flexible routines suited for comparison of different parameterizations of the drift-diffusion model within different experimental designs. For the purposes of the present study we restricted the drift-diffusion model in DMAT to the EZ-diffusion model [cf. Equation (1)], set the outlier treatment to a simple check for whether reaction times exceeded 1.25 s and fitted data from different experimental conditions independently. Even though we used the EZ-diffusion model in this DMAT analysis, the fitting procedure still differed from the EZ method as described in the paragraph above. We obtained parameters of the Bayesian model from the optimized DMAT parameters as described for EZ above.

#### MCMC

For fitting the Bayesian model directly to accuracy and reaction times we used stochastic optimization based on a MCMC procedure. We defined a Gaussian model of the residuals between observed accuracy and reaction time and the model predictions given a specific parameter set. In particular, we defined the log likelihood of the data given all parameters θ = {σ, $\hat{\sigma }$, *T*_*nd*_} as

(43) $$\begin{array}{l} \log p(\text{Acc},RT|\theta )=\log p(\text{Acc}|\theta )+\log p(RT|\theta )\propto \\ \;-w_{\text{Acc}}{\left(\text{Acc}-\text{Acc}(\theta )\right)}^{2}-w_{RT}{\left(RT-RT(\theta )\right)}^{2} \end{array}$$

where Acc is the accuracy observed experimentally, Acc(θ) is the accuracy predicted by the Bayesian model with parameters θ, *RT* is an observed reaction time and *RT*(θ) is that predicted by the model. The weights *w*_Acc_ and *w*_*RT*_ correspond to the expected precisions of the observed measures (see below for the approach of setting these).

Following Philiastides et al. (2011) and as done in DMAT we chose to represent the reaction time distributions of correct and error responses in terms of seven quantiles (0.02, 0.05, 0.1, 0.3, 0.5, 0.7, and 0.9). Consequently, the complete log likelihood function was

(44) $$L(\theta )\propto -w_{\text{Acc}}{\left(\text{Acc}-\text{Acc}(\theta )\right)}^{2}-\sum_{e=0}^{1}\sum_{i=1}^{7}w_{q_{e,i}}{\left(q_{e,i}-q_{e,i}(\theta )\right)}^{2}$$

where *q*_*e,i*_ is the *i*th of the seven quantiles for either correct or error responses as indicated by *e*.

To evaluate the log likelihood function we estimated the predictions of the model for a given parameter set. We did this by simulating the experiment of Philiastides et al. (2011) with the Bayesian model, i.e., for a given experimental condition in one half of the experiment (see description of experiment above) we simulated 1100 two-alternative forced choice trials resulting in a set of 1100 decisions and reaction times from which we computed accuracy and reaction time quantiles. Because these estimates were noisy, we repeated the simulation 30 times and used the average values as estimates of Acc(θ) and *q*_*e,i*_ (θ).

Repeating the simulation 30 times also allowed us to estimate how much the observed accuracies and reaction time quantiles would vary, if the experiment was repeated. These estimates then determined the weight *w*_Acc_ and the 14 quantile weights*w*_*q*_*e,i*__. Before fitting, we set the weights to the inverse variance over 30 repetitions for a particular parameter set which we knew to exhibit a relatively large spread of values (providing an upper bound on the expected variability and resulting in weights: *w*_Acc_ = 4246, mean of the 14 *w*_*q*_*e,i*__ = 3.78). We did not update the weights during fitting, because they represent an estimate of the precision of the experimental observations and not of the precision of the model predictions.

For the simulations we chose Δ*t* = 28.3 ms which provided us with sufficient resolution with only 45 time steps until the outlier threshold at 1.25 s was crossed.

For simplicity we further used wide (effectively uninformed) Gaussian priors over the model parameters (cf. final estimates in Figure 3):

(45) $$p(\sigma )\sim N(0,400^{2})\;p(\hat{\sigma })\sim N(0,400^{2})\;p(T_{nd})\sim N(0,10.2^{2}).$$

The Gaussian priors over standard deviations σ and $\hat{\sigma }$ translate into positive-only priors for the corresponding variances. Although the prior over non-decision time *T*_*nd*_ is also defined for negative values we subsequently restrict values to be positive using a corresponding setting in the sampling method we used. This method was DRAM of Haario et al. (2006) (Matlab mcmcstat toolbox available at http://helios.fmi.fi/~lainema/mcmc/) which is based on Metropolis-Hastings sampling and computes an approximate posterior parameter distribution $\hat{p}$(θ|Acc, *RT*). We initialized the Markov chain at σ = $\hat{\sigma }$ = 2, *T*_*nd*_ = 0.42 *s* and ran it for 3000 samples. We ignored the first 1000 samples (burn-in period) and selected every tenth sample to avoid dependencies within the Markov chain leaving us with 200 samples to estimate the parameter posterior. We visually confirmed that the posteriors were unimodal and approximately Gaussian. Finally, we report the mean and standard deviation of the samples from these posteriors (cf. Figure 3).

**Figure 6:**
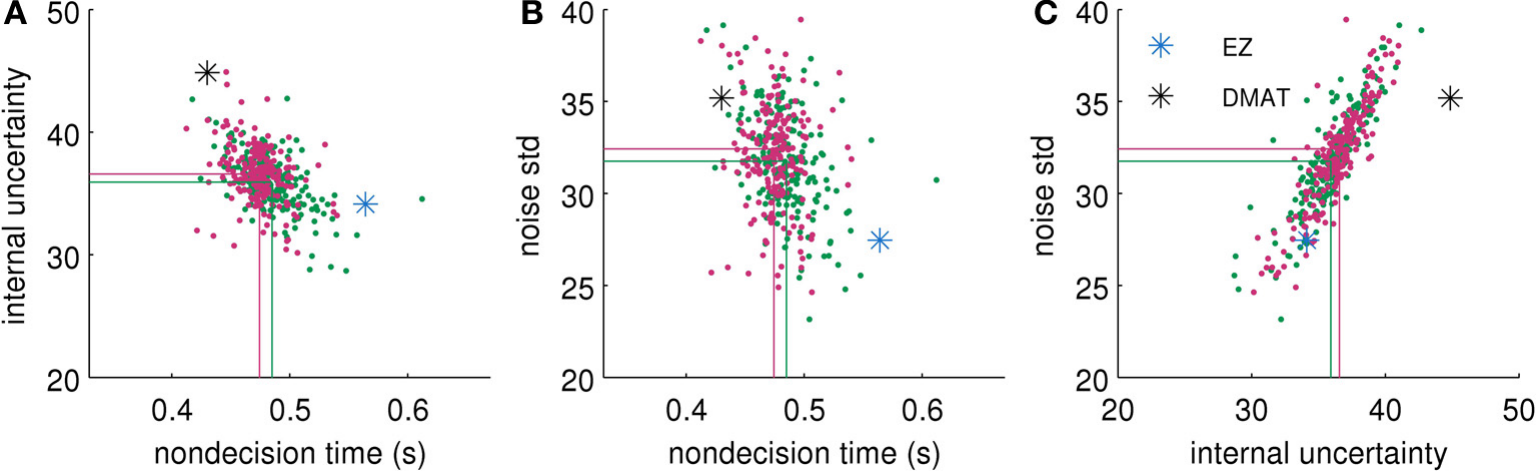
**Comparison of approximate posterior distributions for different parameter priors**. We show two-dimensional scatterplots of samples for each possible pair of parameters [**(A)** non-decision time *T*_*nd*_ vs. internal uncertainty $\hat{\sigma }$, **(B)** *T*_*nd*_ vs. noise standard deviation σ, and **(C)** $\hat{\sigma }$ vs. σ]. Green dots show samples from posterior with priors reported in Equation (45). Red dots show samples from posterior with double as wide priors. Lines visualize the corresponding means over samples. For comparison we also plot the parameter values found by EZ (blue star) and DMAT (black star). The fitted data were obtained from the “low evidence,” SHAM condition of the second half of the experiment of Philiastides et al. (2011). This specific condition showed the largest differences between posteriors (using the two different Gaussian priors), i.e., for all other conditions, we found smaller and often vanishing differences.

To check any influence of the priors on the posterior distribution we repeated the procedure with priors which were double as wide as those in Equation (45). The resulting posteriors (see Figure 6 for an example) differed only slightly from the posteriors reported in Results. In particular, the differences had no effect on the interpretations above.

The described fitting procedure was used as a first approximation to obtaining full parameter posteriors for the Bayesian model. We also found that the stochastic optimization implemented by the MCMC procedure handles the noisiness of our objective function [Equation (44)] well. More refined approaches to this problem have been suggested (e.g., Turner and Zandt, 2012) and we will consider these for future applications.

### Conflict of interest statement

The authors declare that the research was conducted in the absence of any commercial or financial relationships that could be construed as a potential conflict of interest.
